# Climate change mitigation and workers’ interests: why framing a Green New Deal as redistributive and security-enhancing is key to popularity

**DOI:** 10.12688/f1000research.160684.2

**Published:** 2026-01-13

**Authors:** Kevin Ardron, Graham Stark, Sophie Meller, Howard Reed, Matthew Johnson, Elliott Johnson

**Affiliations:** 1 Social Work, Education and Community Wellbeing, Northumbria University Department of Social Work Education and Community Wellbeing, Newcastle upon Tyne, England, NE7 7TR, UK

**Keywords:** Green New Deal, public policy, Public opinion, redistribution

## Abstract

**Background:**

There is urgent need for comprehensive climate change policies to mitigate impacts and protect the interests of those most vulnerable to its worst effects. The Labour Government has rejected its own 2021 £28bn annual investment in climate change policies on account of commitment to economic restraint and public opinion. Not only does this pose real risks to the UK’s ability to respond to climate change, it may also reduce a range of social and economic benefits.

**Methods:**

We report findings of innovative mixed-methods survey analysis of public perceptions of an illustrative Green New Deal within three surveys (1) n=693; 2) n=10; 3) n=2,200) of adult UK residents conducted between November 2023-January 2024.

**Results:**

We analyse the findings of survey 3 to show that levels of support for a Green New Deal are high across parties and demographic groups, and increase further when voters are presented with narrative justifications adversarially co-produced with opponents – termed ‘haters’ – of the policy. We find clear associations between risk of destitution and various other socioeconomic characteristics, health status and levels of support. We present innovative Structural Equation Modelling (SEM) of these associations and find moderately strong positive correlations with levels of support for key infrastructural policies.

**Conclusions:**

This article presents further evidence in support of the notion that exposure to risk of destitution, which varies by age, is a key determinant of policy preference at a time in which political affiliation is increasingly fluid and the prima facie need for a Green New Deal is considerable. This suggests that, in order to understand preferences and to present responses to challenges, there is good reason to focus on material outcomes. Given the importance of a Green New Deal to enhancing financial security, progressive politicians have every reason to commit to substantive reform.

## Introduction

The issue of climate change is a recognised priority for governments globally. The risk to human life from the status quo of carbon intensive industries is existential. Mitigating that risk requires a transformation on the same scale as the New Deal programme which reorganised US society between 1933-1938. It is for this reason that many proponents have called for a Green New Deal policy programme grounded in reshaping the economy in such a way as to ensure sustainability. This whole of government approach directs public resources to achieving net zero via decarbonization and expansion of energy, reduction in energy use, regeneration of biodiversity and reduction in waste. This constitutes a whole-scale shift in priorities that have dominated economies for decades. That shift has invoked opposition from stakeholders with an interest in fossil fuels and the population more broadly where the costs of behavioural change are presented as falling on individuals. However, there is also evidence that an ambitious programme of reform on this scale is required, not just to overcome the challenges of climate change, but to address the UK’s long-term issues of growth, productivity, poverty and inequality. While decarbonisation necessarily requires the loss of jobs in, say, oil fields, the creation of other jobs is essential and those jobs can be organised in such a way to protect the interests of those moving out of fossil fuel-based employment. The sheer scale of investment and the essential role of the state in designing and controlling a Green New Deal opens up scope for directly reducing poverty and inequality by investing in specific communities and setting pay rates at higher levels.

The current Labour Government originally committed itself to a minimum annual investment of £28bn within the Green Prosperity Plan in 2021 ( Labour Party, 2021). This commitment has, however been reduced consistently over time. Instead of investing in a fast-paced transition to renewables, the Government has instead adopted commitment to a Scotland-based clean energy generation company funded at far lower level - £8.3bn over 5 years ( Beament, 2024). This has been justified as a cheaper means of avoiding disruption and job losses in North Sea Oil ( The Herald, 2024) associated with a sudden shift from carbon production, but one that has been criticised for being inadequate to deliver energy security ( Yahoo, 2024). However, inadequate shift to net zero that fails to deliver either on climate change or national security would also fail to provide long-term investment in jobs and the very prosperity that was an original selling point of a Green New Deal. Given that there are critical reasons to achieve net zero, there are grounds to understand whether an adequate policy is socially feasible.

A growing body of evidence indicates that material insecurity drives progressive material policy preferences that reduce poverty and inequality ( Stark, 2024; Stark *et al.*, 2025b; E. A. Johnson *et al.*, 2025a; Hudson *et al.*, 2025; Nettle *et al.*, 2025; Littlefair *et al.*, 2026). In this context, an adequately funded Green New Deal has the potential to be popular – potentially more popular than an inadequately funded policy, even if it incurs public debt, by virtue of its enhancing material security via job creation, energy cost stabilisation and climate change mitigation. This is of growing concern electorally as progressive parties committed to climate change mitigation fall further behind Reform UK, which is opposed to such policies ( Partington, 2025; Murphy & Massey-Chase, 2022; Birkett, 2022). If progressive parties do not commit to an adequate Green New Deal because they believe that doing so is unpopular, evidence that shows the opposite would seem to be reason for parties to present the policy as being in direct contrast to right wing policies that will likely exacerbate longer-term material insecurity associated with climate change. Progressive parties’ ability to demonstrate their policies’ impact on people’s material security underpinned historical success during the New Deal era in the United States and during the UK Labour Party’s programme of post-war rebuilding from 1945-1951. As such, an adequate Green New Deal that secures jobs and energy supplies and mitigates climate change insecurity would seem to be a key potential cornerstone of a successful programme for progressive government.

However, there is a body of evidence that indicates that intensely opposed minorities exert disproportionate influence over policy outcomes relative to their numerical size. Olson’s (1965) foundational work on collective action explains this asymmetry: groups bearing concentrated costs from policy change face strong incentives to organise opposition, while those receiving diffuse benefits lack equivalent motivation to mobilise support. The result is that small, intensely motivated groups can successfully block reforms favoured by numerical majorities ( Lohmann, 1998).

This dynamic operates through multiple mechanisms. Politicians perceive intense opposition as electorally threatening, leading them to avoid or abandon initiatives even when polls indicate majority support ( Burstein, 2003). Research on legislative behaviour confirms that representatives weight opinion intensity alongside opinion direction when calculating political risk ( Page and Shapiro, 1983). Tsebelis’ (1995) veto player framework further demonstrates how institutional structures amplify minority blocking power, enabling determined opponents to obstruct change at multiple decision points. The implications for progressive policy are significant. Reforms generating broad but modest benefits – such as environmental regulation or redistributive taxation – face organised resistance from those bearing concentrated costs, while potential beneficiaries remain politically quiescent ( De Bruycker, 2019). Overcoming this asymmetry requires either mobilising latent support through compelling narratives or designing policies that mitigate opposition intensity by addressing opponents’ core concerns directly.

In this article, we develop an archetypal Green New Deal and test its popularity. We report findings from a series of mixed-methods surveys conducted between November 2023 and January 2024 examining the nature and fluidity of public perception of transformative Green New Deal reform. We first deployed an online screening survey (n=693) using a Prolific panel to identify opponents of the policy, before working with 10 policy ‘haters’ who reported ≤20 levels of support for tax reform to adversarially co-produce ( Johnson, Johnson and Nettle, 2022) four narratives thematically organised around absolute gains, relative gains, security and environmental benefit to persuade people like them to support the policy. We then conducted a nationwide Prolific survey (n=1988) which assessed public support for the policy on the basis of a description of the policy and its impacts and then used a randomised adversarially co-produced narrative as a treatment and collected a range of demographic, socioeconomic and health data for analysis of associations. We outline a series of findings from these data that suggest high levels of support for progressive Green New Deal reform overall, particularly where burdens are placed on wealth and business, significant impact from narratives, particularly on ‘haters’, and clear associations between risk of destitution and various other socioeconomic characteristics, health status and levels of support. We present Structural Equation Modelling (SEM) of these associations. We also report moderately strong positive correlations with levels of support for key infrastructural policies.

### What do people say they want from a large-scale climate change mitigation measures and why?

The majority of people are aware of the problem of climate change, feel concerned about its potential consequences and generally support the notion of mitigation ( Fairbrother, 2022). However, to mitigate impacts of climate change there is need for substantive economic and social transformation globally and within societies. This entails threat to those interests that are grounded, in particular, in fossil fuels and related industries ( Brügger, Morton and Dessai, 2015; Douenne and Fabre, 2020). When *those* material interests are made salient to the broader population, including via potential increases in taxation, public opposition increases, raising an electoral obstacle to urgent reform ( Fairbrother, 2022). In this instance, people are less likely to support measures that offer no short-term benefit, but which pose a threat to financial security via increases in tax or costs of goods. The foreshortening of interests in this context has been described by Botzen as ‘myopic bias’ ( Botzen, 2022). The author identifies a series of further biases that contribute to rejection of climate change policies including simplification of risks as low probability/high consequence, such as flooding ( Robinson and Botzen, 2019), availability of evidence, finite pool of worry against a number of other and more pressing crises and herding of views, each of which intersect with social inequalities and influence the way in which messages about climate risks are received and interpreted. These biases contribute to obstructive social norms that impede accurate assessment of climate risk ( Frederiks, Stenner and Hobman, 2015; Meyer and Kunreuther, 2017).

However, there is evidence of support for climate change policy when framed in terms of protecting financial security and predictability. This has an implicit element of redistribution baked into it, since as Newell *et al.* ( Newell *et al.*, 2021, p. 2) remind us, such measures ‘account for and contest how climate change is having the most severe effects on those with the least responsibility for causing it, and who, at the same time, are often excluded from decision-making processes regarding responses to the problem’. Importantly, there are two distinct approaches that offer different impacts on citizens ( Füssel and Klein, 2006): i) mitigation of risk, which attempts to limit change at a global level over several decades with no immediate benefit to those bearing the cost and ii) adaptation to risk, which moderates the adverse effects of unavoidable climate change, while seizing new opportunities that arise, suggesting greater appeal to individuals where impact is localised and evident, offering a more immediate and tangible return on investment ( Brügger, Morton and Dessai, 2015).

A Green New Deal implies a combination of mitigation and adaptation and Brugger *et al.* suggest that redistributive strategies bridge the two frameworks and serve to enhance popularity by foregrounding the interests of those most vulnerable to climate change ( Brügger, Morton and Dessai, 2015). They argue that emphasising clearly the impact on workers’ interests of climate change risks and responses is particularly important. Moreover, there is evidence that, when policies are framed effectively by those who oppose them, narratives can overcome or reduce biases by making interests salient ( Johnson, Johnson and Nettle, 2022; Johnson *et al.*, 2023). In what follows, we test perception of a Green New Deal designed in such a way as to redistribute and support economic interests of the vast majority of Britons.

### Overview of reforms

The challenge of achieving net zero is considerable, but has clear economic and social rationale ( Millard, 2023). The UK’s economy is in its worst state since 1945 and requires large-scale public investment. Those jobs associated with fossil fuels are time limited, since the cost to society of using fossil fuels increases perpetually as its impacts are felt. Our programme starts from the premise that a whole of Government approach is required, such that departmental budgets ought to be seen as complementary and underpinned by the Treasury, rather than being at odds with one another’s priorities.

Fundamentally, we need to reorganise public investment so that improving sustainability is a central goal for all infrastructure projects. This bears a negligible cost. We then need to invest, as Labour promised in 2021, at least £28 billion a year in decarbonising the energy supply and reducing energy expenditure. This requires creation of a National Investment Bank out of the UK Infrastructure Bank and use of the tax system to incentivise investment in green projects alongside ending new licences for fossil fuel projects as well as removing licences for private energy and water companies in order to deprivatise utilities at low cost.

We need a National Building Service to retrofit and install heat pumps, solar panels and insulation in the housing stock, since the private sector has proven unable to do this on the scale required. The cost is consistent with Labour’s 10 year pledge to invest £60 billion in this area. Doing this trains a workforce and takes up much of the slack created by ending fossil fuel operations. However, given to guarantee security for the 260,000 workers ( Emden, Murphy and Gunson, 2020) in carbon intensive industries and avoid repeating the impacts on coalmining communities of the loss of that industry in the 1980s, we propose a ‘Quadruple Lock’: a) a job guarantee to ensure that those who lose jobs to decarbonisation are employed under the same or better conditions in their region; b) locating investment in areas affected by the shift; c) vocational training to upskill workers; d) providing funding to workers to set up businesses using their existing skills ( Common Sense Policy Group, 2024, pp. 72–73).

We then need to improve and enforce regulations on pollution, divert subsidies away from intensive agriculture toward regeneration at a cost of £5.6 billion per annum over 10 years (full use of a £56bn shortfall in regeneration investment) and protection of biodiversity and charge producers for waste at a cost of £1m per protected site. including via Extended Producer Responsibility (EPR). This raises £1bn per annum. To support such a programme, we require a carbon tax on producers that is progressively increased over time. To mitigate climate change contributing economic activity and achieve net zero we introduce a carbon tax of £55 to £60 per tonne in 2024, rising to £75 per tonne in 2030 and a permanent excess tax on fossil fuel companies combined with a redirection of current subsidies to fossil fuel producers ( Burke *et al.*, 2020). This raises £13.7 bn per annum. We complement this by reversing the cut to fuel duty, effectively returning the UK to the level it would have had if Fuel Duty had risen in line with inflation since 2010 ( Office for Budget Responsibility, 2023). This raises £19.9bn.

Public investment carries with it a multiplier effect of 2.91. Each year of Labour’s original commitment to a £28 billion annual investment would create £77 billion. The US Inflation Reduction Act illustrates the basis of this impact, with 170,000 jobs created, renewable electricity generation increased, inflation reduced much more quickly and growth much higher than in the UK ( Milman, 2023). Given that a Green New Deal performs a similar function, and given that policymakers have focused on the economy as the basis for justifying policies, there is good reason to assess public opinion in terms of the policy’s instrumental value.

## Methods

We followed the methods outlined in Johnson, Johnson and Nettle ( Johnson, Johnson and Nettle, 2022; Johnson *et al.*, 2023), which we outline under each survey description below. These included adversarial co-production of narratives with opponents of policies to persuade people like them to support the policies. The narratives were then presented to a larger group of participants to establish levels of support for policies pre- and post-presentation of narratives. ‘Red Wall’ constituencies are those in the North and Midlands of England and parts of Wales that were traditionally Labour voting but switched to, or came close to switching to, the Conservatives. They played an important role in the outcome of the 2019 General Election and voters in those areas therefore received significant attention from political parties ( MacKinnon, 2020; Kanagasooriam and Simon, 2021; Johnson, Johnson and Nettle, 2022). We therefore engaged with residents of those constituencies in narrative adversarial co-production and ensured that residents were represented within the final survey sample. Our main confirmatory predictions were that subjective economic status and other socioeconomic characteristics will be correlated with levels of policy approval and that people who are firm opponents of policies will produce narratives that can persuade individuals with similar demographic characteristics to support the policy. In exploratory analyses, we examined, whether the four types of narrative for each policy differ in persuasiveness according to the key element (e.g. Absolute gains) around which each narrative will be shaped, whether such narratives have different effects on people based on demographic characteristics or political perspectives, whether there are correlations between levels of support for categories of policies presented and whether levels of support for policies increase overall as additional information on policies are presented. There were three survey stages.

### Survey 1: Screener to assess initial level of support for policies

We conducted a 15-minute screening survey with 693 participants with Red Wall constituency postcodes or, due to platform limitations, area postcodes with a large proportion of Red Wall constituencies on Prolific, an online survey panel that provides researchers with access to participants for a fee. Prolific holds no licence over the data that those participants provide within surveys. We presented brief, bullet-point outlines of proposals for a Green New Deal and asked people to rate them on a 100-point sliding scale in which via a horizontal slider anchored with 0 = strongly disagree and 100 = strongly agree. This elicited very basic popularity data to enable identification of firm opponents of utilities reform for adversarial co-production of narratives. Participants received £2 in compensation.

### Survey 2. Adversarial co-production of narratives

We identified 10 Red Wall opponents (≤20 levels of support for a Green New Deal reform) from the screening survey and invited them to develop narratives that elicit features of the policies to persuade voters like them of its merits. Participants produced written narratives (minimum 150 words) that we standardised for language, style and length (150 words + 10 per cent max). We co-produced four narratives around the most cohesive ideas expressed, using the text provided by participants to organise prose around four specific justificatory elements: 1) absolute gains – the impact of reform on policy that affects all members of society; 2) relative gains – the impact of reform on improving the interests of low-middle income voters at the expense of wealth voters; 3) security – the impact of reform on securing society; 4) biodiversity.

Narrative 1: Absolute Gains: A Green New Deal just makes plain sense for all of us. By transitioning to renewable energy sources like solar, wind and hydro and curbing oil and gas extraction, we can safeguard our planet from the devastating impacts of pollution, extreme weather events, and rising sea levels. And going big in investing to build up renewable infrastructure could kickstart an economic renaissance across the country. This is a real chance to commit to an environmentally sustainable future, where we aren’t threatening our shared future in Britain. I know change on this scale won’t come easy or cheap for that matter. But looking at the shape we’re in today, and thinking about the country and world I want my children and grandchildren to inhabit, pushing hard for renewables just feels like the right call. Communities and populations should own the energy companies. The energy created in Britain is our natural resource and should not be a burden on the shoulders of people.

Narrative 2: Relative Gains: A Green New Deal is about ensuring that energy and prosperity isn’t hoarded by the wealthy few, but shared equitably. This policy not only addresses environmental concerns, but also stimulates economic growth, particularly in less wealthy regions. We can tax wealthy fossil fuel producers and use the funds to develop greener energy infrastructure and jobs on a massive scale, simultaneously reducing income inequality and controlling energy prices. We’re talking about creating stable, good-paying jobs for those who need them most. This will ensure that our most vulnerable people and young children are not left behind in harmful environments. By reducing carbon emissions, we can reduce the impact of climate change globally and ensure more opportunities for countries around the world to expand their means of generating income and becoming wealthier, thereby allowing citizens of those countries to lead healthy and productive lives without having to move elsewhere to earn a living.

Narrative 3: Security: A Green New Deal would provide us with energy security and ensure we are not at the mercy of world events or foreign powers. Because the world only has a limited amount of fossil fuels, those countries that own them hold power over those that need them. As recent conflicts in Ukraine and the Middle East have shown, countries can use their ownership of fossil fuels against us for political gain, reducing supply and increasing prices. Owning our own renewable energy sources will secure us against international events and enable us to get ahead of other states. It will also enable us to develop greener energy production and infrastructure on a massive scale before the costs of non-renewables become prohibitive and cause supply problems which could lead to tensions between states and countries. Regeneration is needed not just for securing our future in an insecure world, but for securing future generations as well.

Narrative 4: Biodiversity: Climate change is real and we need to try our best to make changes to protect species on the planet. Nature is valuable on its own terms and our ecosystems are vulnerable and sensitive. When individual species die off, other species are affected. Threats to nature in Britain come from forms of intensive industry, agriculture and infrastructure that need to be regulated more effectively. We need safe spaces for natural British environments and species to regenerate. The Green New Deal is extremely important because there will be a massive shift in the numbers and diversity of animals surviving and thriving. We can reinvest savings and profits from transitioning to renewable sources of energy into regenerating natural environments in the countryside and at sea. By creating those safe spaces, a Green New Deal would enable populations to recover and ecosystems to become more resilient, ensuring that biodiversity and species are protected.

Participants received £5 remuneration for adversarial co-production.

### Survey 3: Public opinion assessment

A final, 30-minute public opinion survey was conducted between 20-26 January 2024 with 2,200 adult UK residents via Prolific. To ensure effective representation among Red Wall constituents, we first opened the survey to 916 residents with Red Wall postcodes or area postcodes with a large proportion of Red Wall constituencies. We then opened the survey to a further 1,305 participants across Britain. Participants were presented with a description of a series of areas of tax reform as one of ten policy areas welfare reform, Green New Deal, public utilities, health and social care, childhood and early years, education, housing, transport, democratic reform, taxation, along with the impacts such reforms evidence indicates will follow from their implementation ( Stark 2024; Stark *et al.*, 2025a; E. A. Johnson *et al.*, 2025a, 2025b; Hardill *et al.*, 2025; Hudson *et al.*, 2025; Nettle *et al.*, 2025; Littlefair *et al.*, 2026). The description for a Green New Deal was:

A Green New Deal would restructure our economy by:

• converting our energy supply to renewable sources of electricity (wind, solar, nuclear, etc.) through publicly owned energy services
• ending new licences for oil and gas extraction
• creating jobs in renewable sources of energy
• protecting workers by guaranteeing jobs in publicly owned energy companies
• introducing new legislation to improve air and water quality and making producers pay for waste and disposal
• publicly funding natural regeneration rather than intensive agriculture and introducing marine protected areas

Evidence suggests that impacts of the policies include:

• Protecting all citizens from the worst effects of pollution, extreme weather events, including heat waves, flooding, drought and rising sea levels
• Increasing the spending power of those on low-medium incomes by using tax on wealthy individuals and companies to create infrastructural jobs in less wealthy regions and control energy prices
• Regenerating natural environments by increasing biodiversity on land and sea
• Reducing unplanned migration from areas of the world that become less habitable due to climate change

Participants were asked to rate their opposition or support to those policies on a scale of 0-100. They were then shown a randomised adversarially co-produced narrative and asked to rate its persuasiveness on a scale of 0-100 and then to rate their opposition or support for the policy again on a scale of 0-100. Participants were then asked to provide basic demographic data, socioeconomic data, including self-rating status on the MacArthur ladder of subjective socioeconomic status ( Adler *et al.*, 2000), and perceived risk of destitution on a 100-point sliding scale, health status, including Depression PHQ-8 ( Kroenke, Spitzer and Williams, 2001), Anxiety GAD7 ( Spitzer *et al.*, 2006), single item life satisfaction ( Guney, Kalafat and Boysan, 2010; Mamani-Benito *et al.*, 2022) political affiliation, voting intention and faith in politicians established by six items in prior project iterations ( Johnson *et al.*, 2023). Participants received £4.50 in remuneration.

### Data analysis

Data were analysed using Julia. Relative to General Election voting intention in January 2024, our sample overrepresented people who voted as compared with not voting, overrepresented Labour voters and underrepresented Conservative voters (see Table 1 below). In the statistical analyses that follow, we have therefore applied post-stratification weights that make our sample representative of age and contemporary voting intention as of 26 January 2024 ( Politico, 2024). The election result itself showed: Labour 33.7%, Conservatives 23.7%, Reform 14.3%, Lib Dems 12.2%, Greens 6.4%, SNP 2.5% and Plaid 0.7%.

**Table 1.** Political preferences.

Political Party	Voting in 2019	Intention for 2024
Conservative	30%	13%
Labour	30%	24%
LibDem	7%	5%
Nat/Green	6%	5%
No Vote/DK/Refused	21%	46%
Other/Brexit (Reform in 2024)	6%	6%

**Table 2.** Demographic and socioeconomic statistics.

Variable	Mean	Median	Standard Deviation
Age	47.96	48	16.74
Left Right	45.97	50	21.01
Household Net Income Pa	40,156.70	34,000.00	39,420.12
Perceived Risk Of Destitution	26.85	19	26.86
MacArthur Ladder Score	5.3	5	1.61
Perceived Control Of Life	61.41	65.17	22.69
Life Satisfaction Score	61.64	68	24.15
GAD-7	5.14	4	5.2
PHQ-8	4.45	3	4.96

Not included in the Politico polling were NI parties 2.5% and independents 2% ( Baker, Pollock and Cracknell, 2024). The final result reflected low turnout among those who indicated Labour as a voting intention, possibly because they were likely to be younger and younger people are less likely to vote overall and also because polling leads suggested a decisive Labour victory and reduced competitive pressure to avoid an alternative outcome.

Categorical variables were contrast coded, and continuous variables scaled. The distribution of residuals for all models was satisfactory. All p values are two sided. Our confirmatory predictions were that those who strongly rejected a Green New Deal would be homeowners, express low risk of destitution on scale of 0-100 (<30) and intend to vote Conservative in 2024. The rest of the analyses are considered exploratory.

We followed our established method ( Johnson *et al.*, 2023) of structural equation modelling (SEM) in R package ‘lavaan’ ( Rosseel, 2012) to estimate covariance between socioeconomic position (measures above), mental health (measures above), faith in government and age and to regression relationships between those variables and support for a Green New Deal. On the basis of our prior studies ( Johnson, Johnson and Nettle, 2022), we hypothesised that the latent variables would directly affect support for a Green New Deal, with lower socioeconomic status, higher mental distress, younger age and greater faith in government associated with higher levels of support for the policy as a means of reducing financial insecurity (associated with lower socioeconomic status, higher mental distress, younger age) and as a possible means of improving society (associated with greater faith in government).

Our raw data, Julia scripts and R scripts are publicly available ( Johnson, 2024; E. Johnson *et al.*, 2024a; Stark, 2024).

## Results

### Participant demographic characteristics

Key demographic and socioeconomic sample characteristics are outlined in Table 1. The sample included 51% female, 48% male and 1% who described themselves in another way. 85% of respondents identified as white, slightly higher than in the 2021 England and Wales Census (81.7%), and 15% identified as belonging to other ethnic groups, slightly lower than the same Census (20.3%), while the median age was 48.00 (mean 47.96, s.d. 16.74), higher than in the 2021 England and Wales Census ( Office for National Statistics, 2022). The median annual non-equivalised household income was £34,000, higher than the national median income for the year ending 2023 of £32,500 ( Department for Work and Pension, 2024). Participants reported a mean score of 26.85 for risk of destitution, with 0 representing extremely low risk and 100 extremely high risk. The mean MacArthur ladder score was 5.30, with 1 representing the worst off in society and 10 the best off. The mean average control of life score was 61.41, where 0 means completely out of control and 100 means completely in control. The mean life satisfaction score was mean 61.63, where 0 means completely dissatisfied and 100 completely satisfied. The average GAD-7 score fell within the 5-9: Mild Anxiety category ( Sapra *et al.*, 2020). The average PHQ-8 score fell below the minimum threshold for depression ( Kroenke *et al.*, 2009).

Participants indicated that they sat broadly in the middle of a left-right 100-point ideological scale. As Table 1 indicates, the sample under-represented both Conservative and Labour 2019 voters, with the proportion of undecided 2024 voters much higher than in national polling. The sample showed a reduction in levels of support for both main political parties.

### Levels of support

Pre-treatment level of support for a Green New Deal was high (mean 70.44, median 77.00, s.d. 25.97). A large proportion of respondents – whom we term lovers – expressed strong pre-treatment support (≥70, 58.33%). A small proportion – haters – expressed strong opposition (≤30, 8.27%). 15.13% chose 100 on the scale, while just 1.90% chose 0. There were statistically significant differences by voting intention. Taking Conservative voting intention, female and not working as reference categories (70.248, R ^2^ = .137, p < .001, 95% CI [62.277, 78.219]), Labour (18.84, R ^2^ = .137, p < .001, 95% CI [14.938, 22.731]), Liberal Democrats (19.39, R ^2^ = .137, p < .001, 95% CI [12.622, 26.162]), Green/SNP/Plaid Cymru (26.740, R ^2^ = .137, p < .001, 95% CI [21.572, 31.908]), Reform (13.291, R ^2^ = .137, p < .001, 95% CI [5.625, 20.956]) or not intending to vote (6.062, R ^2^ = .137, p = .002, 95% CI [2.170, 9.954]) voting intention associated with higher levels of support for a Green New Deal.

Associations for ethnicity, residency, employment status and household income were not statistically significant. However, older age (-0.206, R ^2^ = .137, p < .001, 95% CI [-0.280, -0.133]) and male gender identity (-3.626, R ^2^ = .137, p < .001, 95% CI [-5.683, -1.570]) were predictors of lower levels of support. Dissatisfaction with income (4.471, R ^2^ = .005, p < .002, 95% CI [1.698, 7.244]), risk of destitution (0.095, R ^2^ = .011, p < .001, 95% CI [0.055, 0.135]), high risk of destitution (((≥70, 5.072, R ^2^ = .004, p < .001, 95% CI [1.677, 8.468]) were predictors of lower levels of support, while higher MacArthur ladder score (-0.991, R ^2^ = .004, p = .004, 95% CI [-1.667, -0.315]), home ownership (-4.036, R ^2^ = .006, p < .001, 95% CI [-6.286, -1.785]) and being mostly satisfied with income (-9.352, R ^2^ = .010, p < .001, 95% CI [-14.291, -4.414]), were significant.

While there were differences in self-rated physical health were not significant, higher anxiety (0.402, R ^2^ = .007, p < .001, 95% CI [0.193, 0.612]) and depression (0.449, R ^2^ = .008, p < .001, 95% CI [0.231, 0.666]) scores were associated with higher support.

### Structural Equation Modelling

**Figure 1.**
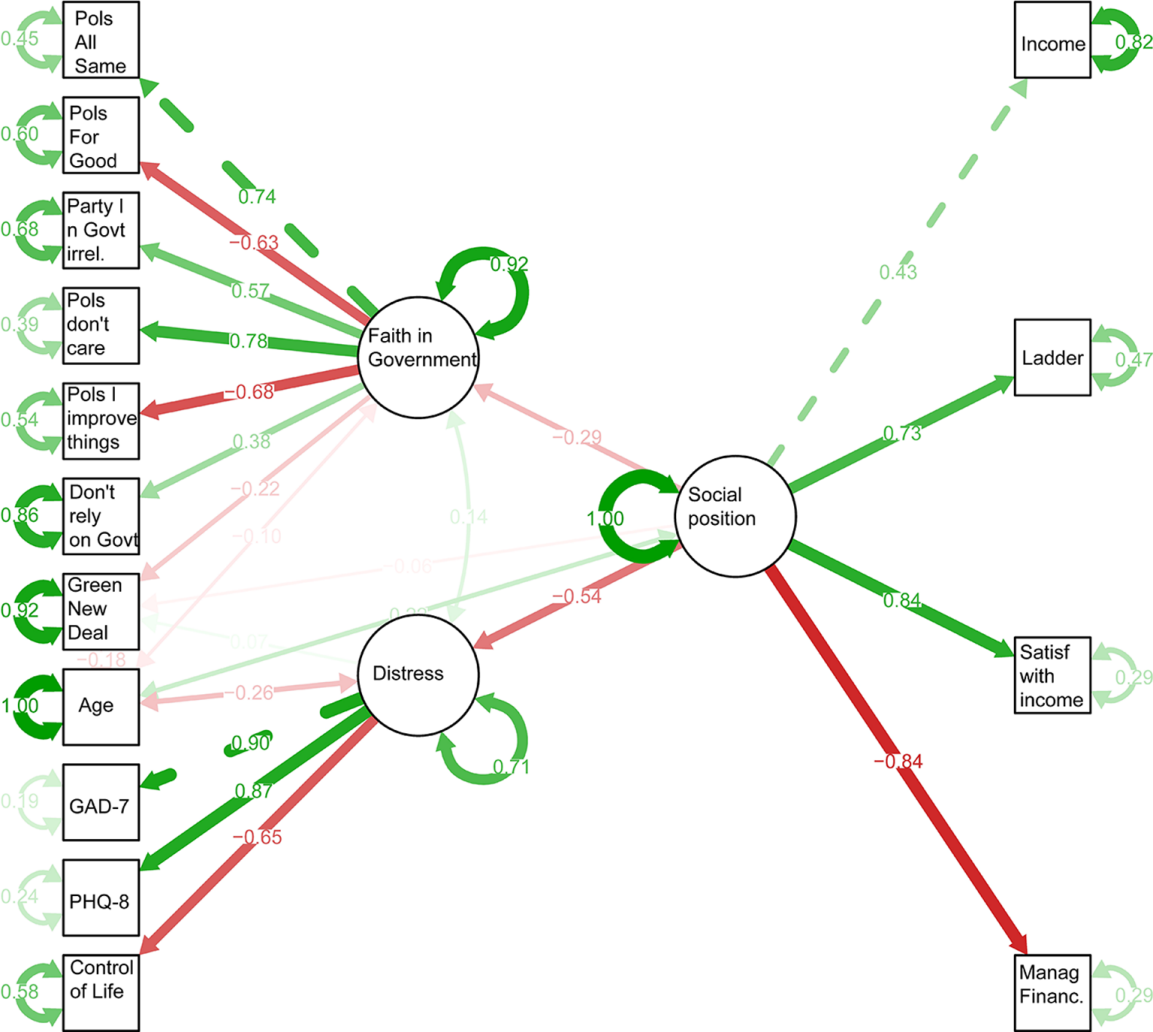
Structural equation model predicting support for Green New Deal. Boxes show measured variables, and ovals inferred latent variables.

As discussed in the Methods section, we fitted the SEM shown in Figure 1 to the data, collapsing across narrative conditions. The full output is provided in Table 3.

**Table 3.** SEM model lavaan package output data.

lavaan 0.6-10 ended normally after 104 iterations	
**Estimator**	ML
**Optimization method**	NLMINB
**Number of model parameters**	38
**Number of observations**	1988
**Model Test User Model:**	
**Test statistic**	969.057
**Degrees of freedom**	82
**P-value (Chi-square)**	0.000
**Parameter Estimates:**	
**Standard errors**	Standard
**Information**	Expected
**Information saturated (h1) model**	Structured

Latent Variables:						
	Estimate	Std.Err	z-value	P(>|z|)	Std.lv	Std.all
**faith_gov =~**						
** Pols_All_Same **	1				0.857	0.744
** Pol_For_Good **	-0.705	0.027	-25.844	0	-0.604	-0.635
** Gov_Not_Matter **	0.864	0.037	23.252	0	0.74	0.57
** Pol_Not_Care **	0.926	0.03	31.045	0	0.794	0.78
** Pol_Want_Imprv **	-0.719	0.026	-27.638	0	-0.616	-0.681
** Pol_Not_rely **	0.501	0.032	15.47	0	0.43	0.379
**soc_pos =~**						
**log_income**	1				0.306	0.429
**Ladder**	3.82	0.214	17.835	0	1.169	0.729
**Satisf_Income**	4.382	0.236	18.554	0	1.341	0.84
**Mang_Financial**	-2.759	0.149	-18.565	0	-0.844	-0.843
**distress =~**						
** sqrt_gad_7 **	1				1.113	0.902
** sqrt_phq_8 **	1.012	0.023	44.657	0	1.126	0.872
**In_Control**	-13.13	0.415	-31.674	0	-14.607	-0.649

Regressions:						
	Estimate	Std.Err	z-value	P(>|z|)	Std.lv	Std.all
**faith_gov ~**						
**soc_pos**	-0.799	0.084	-9.512	0	-0.285	-0.285
**distress ~**						
**soc_pos**	-1.966	0.13	-15.137	0	-0.541	-0.541
** Green_ND ~**						
**soc_pos**	-5.131	2.408	-2.131	0.033	-1.57	-0.064
**faith_gov**	-6.338	0.745	-8.507	0	-5.429	-0.22
**distress**	1.495	0.666	2.244	0.025	1.663	0.067
**Age**	-0.275	0.036	-7.542	0	-0.275	-0.175

Covariances:						
	Estimate	Std.Err	z-value	P(>|z|)	Std.lv	Std.all
**.faith_gov ~~**						
**.distress**	0.106	0.022	4.887	0	0.138	0.138
**.distress ~~**						
**Age**	-3.804	0.374	-10.184	0	-4.065	-0.259
**.faith_gov ~~**						
**Age**	-1.289	0.318	-4.056	0	-1.57	-0.1
**soc_pos ~~**						
**Age**	1.038	0.129	8.014	0	3.391	0.216

Variances:						
	Estimate	Std.Err	z-value	P(>|z|)	Std.lv	Std.all
**.Pols_All_Same **	0.592	0.025	23.64	0	0.592	0.446
**.Pol_For_Good **	0.54	0.02	27.299	0	0.54	0.597
**.Gov_Not_Matter **	1.139	0.04	28.533	0	1.139	0.675
**.Pol_Not_Care **	0.404	0.019	21.639	0	0.404	0.391
**.Pol_Want_Imprv **	0.439	0.017	26.081	0	0.439	0.536
**.Pol_Not_rely **	1.101	0.036	30.495	0	1.101	0.856
**.log_income**	0.416	0.014	30.446	0	0.416	0.816
**.Ladder**	1.206	0.047	25.846	0	1.206	0.469
**.Satisf_Income**	0.749	0.04	18.848	0	0.749	0.294
**.Mang_Financial**	0.29	0.016	18.593	0	0.29	0.289
**.sqrt_gad_7 **	0.284	0.022	12.953	0	0.284	0.187
**.sqrt_phq_8 **	0.4	0.024	16.523	0	0.4	0.24
**.In_Control**	292.508	10.166	28.773	0	292.508	0.578
**.Green_ND**	562.551	18.054	31.159	0	562.551	0.923
**Age**	247.052	7.836	31.528	0	247.052	1
**.faith_gov**	0.674	0.038	17.623	0	0.919	0.919
**soc_pos**	0.094	0.01	9.371	0	1	1
**.distress**	0.876	0.04	22.096	0	0.707	0.707
**Fit Measures**						
**Comparative Fit Index**	0.919					
**RMSEA**	0.074					

The comparative fit index was 0.92; >0.90 is generally taken to indicate adequate model fit ( Bentler, 1990). The root mean square error of approximation (RMSEA) statistic was 0.07; < .05 is generally taken to indicate a very good fit and < .10 reasonable fit ( Fan *et al.*, 1999).

Standardised model parameters are shown in Figure 1. The modelling strongly supports prior work asserting the relationship between socioeconomic status and anxiety and depression ( Parra-Mujica *et al.*, 2023; Villadsen *et al.*, 2023; Nettle *et al.*, 2024) and policy preferences ( Howard *et al.*, 2023, 2024; Johnson *et al.*, 2023). Socioeconomic position affected support for a Green New Deal directly, with those with higher socioeconomic status producing lower support and then indirectly since higher socioeconomic status was associated with lower mental distress, which was also associated with lower support, while lower socioeconomic status was associated with higher levels of cynicism in government, which was associated with lower levels of support. When controlling for socioeconomic status, older age was also associated with lower levels of support. This is partially explained by the association between age and distress, such that older participants were less likely to be distressed.

### Narrative treatment and change in policy approval

**Figure 2.**
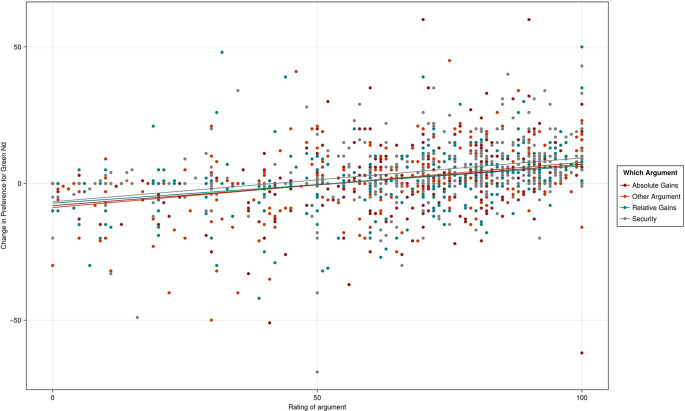
Scattergram of change in Green New Deal preferences from pre-treatment score (< 95).

As Figure 2 shows, there was evidence of a statistically significant narrative treatment effect on support for a Green New Deal on participants overall, which increased by 1.63 points (p < .05) on average. However, as 15.13% chose 100 at the pre stage, there was little room on the scale for improvement among a large proportion of respondents, with a non-statistically significant 0.09-point (p .033) increase in support among pre-treatment lovers, though the percentage choosing 100 at post-narrative stage increased to 17.35 points. There was a larger, but non-statistically significant increase in support among pre-treatment haters of 2.59 points (p.14).

The average change in support by narrative was 1.697 for absolute gains, 0.952 for relative gains, 3.138 for security and 0.648 for biodiversity. Taking absolute gains as the reference category (1.720, R ^2^ = .0.009, p < .001, 95% CI [0.701, 2.739]), the security narrative produced a significantly larger change in support (1.441, R ^2^ = .0.009, p = .030, 95% CI [0.141, 2.753]). The other narratives did not differ significantly from absolute gains. No other associations were statistically significant.

Table 4 sets out average levels of persuasiveness of narratives as rated by participants. There were statistically significant treatment differences in the impact of the narratives. Although the sample size is small and ought to be understood in that context, haters rated, for example, relative gains (mean 17.87, median 15.00) more highly than biodiversity (mean 11.23, median 7.57).

**Table 4.** Average participant-scored persuasiveness of narratives broken down by initial levels of support for policy.

Sample	Absolute gains Mean	Absolute gains Median	Relative gains Mean	Relative gains Median	Security Mean	Security Median	Biodiversity Mean	Biodiversity Median
**All**	68.08	74.00	73.44	81.00	69.61	73.45	63.99	70.00
**Lovers**	82.69	88.00	86.36	91.00	81.99	84.00	77.47	81.00
**Haters**	14.67	8.92	17.87	15.00	19.12	5.50	11.23	9.97

### Levels of support for a Green New Deal compared with levels of support for reform of utilities

Levels of support for a Green New Deal were compared with levels of support for utilities reform – a policy with similar implications on energy security. The description of that policy was as follows:

Reforms to Public Utilities (energy and water) would return water and energy to public ownership:

• buying back the National Grid and British Gas
• taking over water licences
• investing in infrastructure
• managing supplies democratically

Evidence suggests that impacts of the policies include:

• Ensuring that all citizens are protected from power and water shortages
• Increasing the spending power of those on low-medium incomes by using tax on wealthy individuals and companies to control prices and create infrastructural jobs in less wealthy regions
• Securing energy supplies from an insecure international energy market affected by wars and conflicts
• Controlling our energy and water democratically

**Figure 3.**
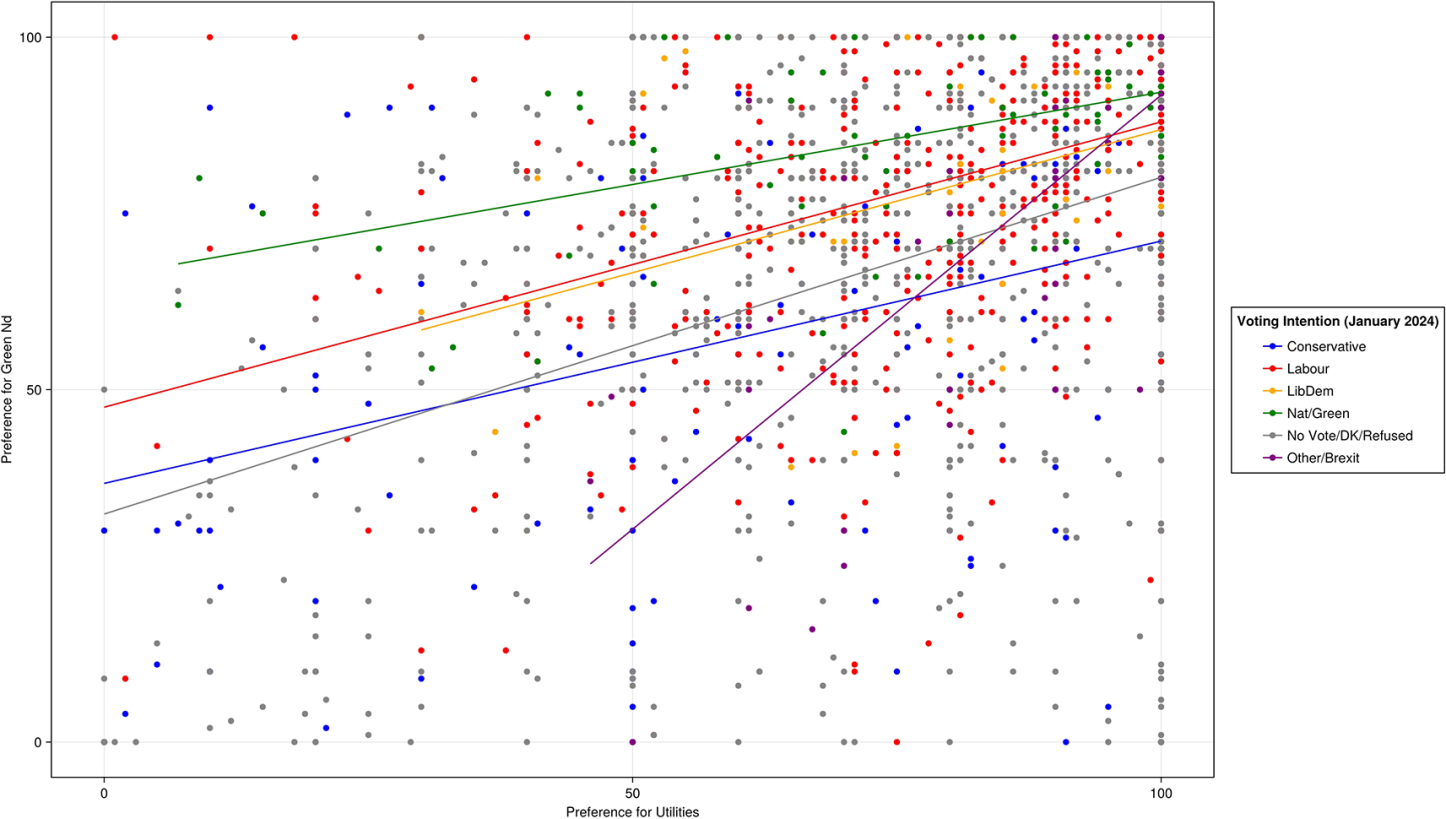
Pre-treatment preferences for a Green New Deal and reform to public utilities by voting intention.

As Figure 3 shows, results suggest that the more individuals support clearly redistributive reforms to welfare and taxation, the more they support a Green New Deal. Support for a Green New Deal was moderately strongly correlated with support for utilities reform (r(1986) = .45, p < .001).

## Discussion

Support for a Green New Deal was extremely high and increased further post-treatment. This concurs with previous findings on redistributive policy ( Johnson *et al.*, 2022) but contradicts claims of widespread public opposition. Support increased via the narrative treatment. The narrative that most emphasized redistribution – relative gains – was most impactful among ‘lovers’ and ‘haters’, indicating support for Brügger *et al.* (2015) claims with regard to the salience of material interests. This is an important finding given the context of budgetary restraint pursued by Government since the 2024 General Election. Increasing taxation on wealth and carbon production to fund an expansive programme of investment such as the Green New Deal outlined in this article or, indeed, Labour’s original £28bn annual investment is likely to be popular, particularly if it is framed in terms of redistribution. We find no evidence to support the assumption that the public are opposed to climate change measures in general or progressive taxation to fund such a programme.

The lower level of support for and impact via the biodiversity narrative may provide further evidence that people’s public policy preferences are influenced more by material circumstances and the prospective impact of policies on immediate, direct outcomes than by abstract values or external interests. This may support a consequentialist understanding of preferences ( Johnson, Johnson and Nettle, 2022). It may be, though, that further connecting people’s interests to biodiversity can enhance support further. Presenting biodiversity as an end in itself, rather than a means to upholding people’s interests is less likely to be successful in advancing a Green New Deal, particularly among ‘haters’.

Third, we find that perceived risk of destitution, in particular, predicts stronger support for a Green New Deal. Part of this association is mediated by psychological distress (depression, anxiety and lack of perceived control). There were strong associations in our data between perceived risk of destitution and psychological distress. This complements recent findings in the Changing Cost of Living Study, which found that increases in financial insecurity have immediate impacts on anxiety and depression ( Nettle *et al.*, 2023). Greater psychological distress in turn increased support for a Green New Deal. Thus, risk of destitution motivates support for progressive socioeconomic policies in part potentially because people recognize that the policy offers means of alleviating distress caused by risk of destitution.

However, greater risk of destitution is also associated with lower levels of faith in government, possibly because risk of destitution is perceived as stemming from failure in policy.

Lower faith in government in turn was associated with reduced support for a Green New Deal, perhaps because people believe that government is incapable of implementing policy in ways that enhance their interests. This supports our previous findings on welfare reform, which indicated that a ‘downward spiral’ of increasing inequality can promote cynicism in ways that foster further inequality ( Johnson *et al.*, 2023). However, as in that article, we find that this negative pathway was more than offset by the propensity of greater risk of destitution to increase support for the progressive policy.

The findings here support the notion of a post-Financial Crisis shift away from small state, pro-private sector thinking toward larger, more interventionist state ( Nettle *et al.*, 2021; Common Sense Policy Group, 2024). The cost-of-living crisis has highlighted the threat to financial security not from increased taxation, but from rising costs of essentials that are currently provided largely by the private sector. This, combined with rising concern about the declining standards in service provision by and increased profit making within business gives rise to decreased support for privatization ( Sloman, 2021).

Progressive parties across Western democracies face a persistent critique: that they have abandoned material concerns affecting working-class constituencies in favour of cultural and identity-based politics ( Dalton, 2018). In the UK context, this narrative gained particular traction following the 2019 general election, with the loss of traditional Labour seats in deindustrialised regions framed as evidence of a fundamental disconnect between progressive politicians and their former electoral base ( Hooper, 2022). A Green New Deal offers a strategic opportunity to recentre progressive politics around tangible economic transformation while simultaneously addressing the climate crisis ( Mazzucato, 2019).

The core insight from the findings presented here is that decarbonisation need not be presented as an additional burden on communities already experiencing material insecurity. Rather, a Green New Deal reframes the energy transition as an industrial strategy capable of delivering secure employment, regional investment, and renewed purpose to areas that experienced the traumatic deindustrialisation of previous decades ( Warren, Stephenson and Wistow, 2021). This matters politically because it shifts debate from the terrain of values and identity—where progressive parties are often defensive—to questions of jobs, wages, and local economic revival, where they can credibly claim to offer concrete improvements ( Valero *et al.*, 2023).

Evidence suggests this reframing resonates with public opinion. Research indicates that when climate policy is presented in terms of employment creation and fair distribution of costs and benefits, support extends well beyond traditional environmentalist constituencies. Polling demonstrates that 63% of ‘Red Wall’ voters support net zero targets, while support for green jobs and energy-efficient infrastructure stands at 75% even in small towns ( Murphy and Massey-Chase, 2022). The challenge for progressive parties has been that climate policy is often perceived as emanating from metropolitan, highly-educated voters whose priorities differ from those in post-industrial regions. A Green New Deal disrupts this perception by explicitly prioritising investment in precisely those communities, positioning renewable energy manufacturing, retrofit programmes, and green infrastructure as successors to the industrial employment that once defined these areas ( Sheridan *et al.*, 2022).

This approach also offers a response to the ‘just transition’ concerns that have emerged as decarbonisation accelerates. Communities in places like Teesside, which have experienced successive waves of industrial decline, are understandably sceptical of promises that this transition will differ from previous experiences of managed decline ( Sovacool *et al.*, 2025). A Green New Deal that guarantees equivalent or better employment, funds retraining, and ensures affected workers have genuine agency in shaping local transitions can begin to rebuild trust that has been eroded over decades ( Upham, Sovacool and Ghosh, 2022).

Crucially, this strategy does not require progressive parties to abandon commitments to equality and inclusion. Rather, it demonstrates that these commitments are compatible with, and indeed essential to, an economic programme that delivers material benefits broadly. The Green New Deal becomes a vehicle for showing that progressive politics is not a zero-sum competition between different constituencies but an agenda capable of improving conditions for the many ( Tagliapietra and Veugelers, 2021). By leading with economic transformation and weaving questions of fairness throughout, progressive parties can escape the framing that positions them as primarily concerned with cultural signifiers rather than people’s lived economic circumstances.

Adversarial co-production of narratives represents a methodological innovation that draws on insights from deliberative democracy and the Narrative Policy Framework to develop policy proposals capable of withstanding opposition. The approach involves systematically engaging with competing frames and counterarguments during the policy development process itself – rather than merely anticipating objections *post hoc* ( Clark *et al.*, 2021).

The underlying logic derives from research demonstrating that exposure to competing narrative frames reduces the influence of any single frame on policy attitudes ( Druckman and Nelson, 2003). When citizens encounter multiple framings of an issue through deliberation, they develop more coherent and considered preferences that prove resistant to subsequent elite-driven reframing attempts ( Barabas, 2004). Adversarial co-production operationalises this insight by building oppositional perspectives into the narrative construction process from the outset.

The method addresses a core challenge identified in narrative policy analysis: that policy narratives typically emerge from coalitions of like-minded actors who inadvertently construct arguments vulnerable to counterframing ( Jones and McBeth, 2010). By contrast, adversarial collaboration – originally developed in experimental psychology to resolve scientific disputes through joint research design ( Kahneman, 2003) – forces proponents to confront the strongest versions of opposing arguments. This produces narratives that have already been ‘stress-tested’ against the objections they will encounter in public debate.

Johnson, Johnson and Nettle have developed this approach into a systematic methodology for progressive policy development, arguing that advocates must ‘break the Overton Window’ by constructing narratives that directly engage with – rather than dismiss – opposition concerns ( Johnson *et al.*, 2023). Their empirical work demonstrates that ‘Red Wall’ constituencies are not inherently opposed to progressive policies such as Universal Basic Income; rather, support depends substantially on the narrative framing employed, particularly when materialist arguments emphasising economic security are foregrounded ( Johnson, Johnson and Nettle, 2022). This finding extends to policies including public utility renationalisation, where adversarial narrative co-production reveals latent support that conventional polling may obscure ( Johnson *et al.*, 2025b). Crucially, their research suggests that transformative policy can achieve feasibility in traditionally sceptical constituencies when narratives are co-produced through genuine engagement with competing perspectives, balancing ambition with electoral pragmatism ( Johnson *et al.*, 2022).

For progressive policy specifically, this approach offers strategic value. Research on deliberative polling demonstrates that participants who engage with diverse perspectives soften strongly held views and prove more receptive to policy change ( Fishkin, 2009; Curato *et al.*, 2017). Narratives developed through adversarial co-production can pre-emptively incorporate the concerns – economic, cultural, or procedural – that might otherwise crystallise into organised opposition. The resulting policy stories speak to multiple constituencies precisely because they have been forged through engagement with genuinely competing perspectives, rather than constructed within ideologically homogeneous advocacy coalitions.

It is important to note, in this context, that a focus on energy security may imply a nationalistic or isolationist approach at odds with broader need for international cooperation to mitigate climate change. One response is that no action on climate change is possible without national governments being able to pursue appropriate policies. The locus of energy security has been national government, with wide divergence even within, for example, the European Union. If parties committed to an adequate Green New Deal are unable to enter and remain in government, then no progress is likely to be achieved, contributing to a downward spiral of climate diminishing policies (Johnson *et al.*, 2024). National progress on addressing climate change is also likely to have broader, positive international effects, as the viability of a Green New Deal is demonstrated. As such, even if focused on national action, the outcomes of increasing support and achieving electoral feasibility are likely to be far preferable to not addressing national-based concerns at all. In this regard, reviewer 1’s reference to Conservative Prime Minister, Margaret Thatcher’s, call for international cooperation and action on climate change is salient ( Roe-Crines, 2025). Having need for action and electoral feasibility established creates pressure on otherwise non-progressive actors to adopt progressive policy. The adversarially co-produced narratives above demonstrate that impact on those affiliated with centre-right and right-wing parties is possible.

## Conclusion

This study demonstrates that ambitious Green New Deal reforms command substantial public support, contradicting assumptions that electoral considerations necessitate modest climate policy. Pre-treatment support averaged 70.44, with nearly 60% expressing strong approval. Adversarially co-produced narratives increased support significantly, with security framing proving most effective overall and relative gains resonating particularly strongly among both firm supporters and opponents. This suggests that emphasising material redistribution – directing resources from wealthy individuals and fossil fuel companies toward job creation and energy price control in less prosperous regions – enhances rather than undermines climate policy popularity. The findings reveal that perceived risk of destitution substantially predicts support for transformative climate action, operating through multiple pathways. Material insecurity directly motivates support while simultaneously generating psychological distress, which further increases policy approval. However, destitution risk also reduces faith in government, which suppresses support – suggesting that progressive parties must demonstrate both ambition and competence to overcome cynicism born of policy failure. Crucially, the positive pathways outweigh this negative effect, indicating that addressing material insecurity through climate investment represents viable political strategy. Adversarial co-production enabled construction of narratives that withstood opposition while appealing across constituencies. The approach demonstrates that engaging systematically with competing perspectives generates more robust policy narratives than advocacy within ideologically homogeneous coalitions. For progressive parties navigating tension between climate urgency and electoral pragmatism, these results suggest that adequately funded Green New Deals framed around job guarantees, regional investment and energy security offer credible means of reuniting progressive politics with working-class material concerns while delivering essential decarbonisation. The question is not whether ambitious climate policy is feasible, but whether progressive parties possess courage to pursue it.

### Ethics and consent

This study has been approved by the Faculty of Health and Life Sciences ethics committee, Northumbria University. The approval number is 5814 and the date of approval was 30 November 2023. This committee contains members who are internal to the Faculty. This study was reviewed by members of the committee, who must provide impartial advice and avoid significant conflicts of interests. Participants provided informed written consent. The study adheres to the Declaration of Helsinki.

## Data Availability

Open Science Framework: What do British residents think about public policy?: Public Opinion Surveys. https://doi.org/10.17605/OSF.IO/3UX4M ( Johnson, M. T *et al.*, 2025). The project contains the following underlying data:
• Full survey dataset • Study protocol • Tax calculations Data are available under the terms of the Creative Commons Attribution 4.0 International license (CC-BY 4.0).
