# Supplementary material for: Climate change mitigation and workers’ interests: why framing a Green New Deal as redistributive and security-enhancing is key to popularity
Source: F1000Res. 2026 Jan 13;14:410. Originally published 2025 Apr 7. [Version 2] doi: 10.12688/f1000research.160684.2 (PMC13000395; doi:10.12688/f1000research.160684.2)
Supplement: Peer review history

# Reviewer report: Reviewer response for version 2

**DOI:** 10.5256/f1000research.194739.r460943

**Published:** 2026-03-18

**Authors:** Leigh Hoath

**Affiliations:** 1 Leeds Trinity University, Leeds, England, UK

This article introduces an area that is pertinent and relevant with currency in the social and political climate we are in. It offers an early challenge to the position in achieving net zero which is underpinned with reading and evidence. 

 The political powers presented within introduction touch upon a number of 'big ticket' concerns around equality and disproportionate dominance. When taking these into account (particularly the reference to the influence of Reform) it might be worth considering the claims around the support within the population of the consequences of climate change and support for related policy. 

 With regards to public opinion - how confident are the authors that they public really understand what is being asked of them? 20% of School Leavers were able to correctly identify the definition of net zero in a large scale survey published at the end of 2024 which begs the question what level of literacy in this area that the general public have. This levies the question of how informed the sample are to be able to make informed decisions creating the narrative presented within this work. 

 It is worth acknowledging how the political landscape has changed since the data was collected - this will position the work more accurately. 

 The study is well constructed with appropriate methodology and research design with the conclusions being drawn coherent in relation to the aims and data collected.

 The findings are compelling and there are obvious next steps which could be taken from this work.

 Overall the article offers a persuasive account of the findings which could bring about a shift in policy.

Is the work clearly and accurately presented and does it cite the current literature?

Yes

If applicable, is the statistical analysis and its interpretation appropriate?

Yes

Are all the source data underlying the results available to ensure full reproducibility?

Yes

Is the study design appropriate and is the work technically sound?

Yes

Are the conclusions drawn adequately supported by the results?

Yes

Are sufficient details of methods and analysis provided to allow replication by others?

Yes

Reviewer Expertise:

Education

I confirm that I have read this submission and believe that I have an appropriate level of expertise to confirm that it is of an acceptable scientific standard.

# Reviewer report: Reviewer response for version 1

**DOI:** 10.5256/f1000research.176615.r425581

**Published:** 2026-01-02

**Authors:** Mary Robertson

**Affiliations:** 1 Queen Mary University of London, London, England, UK

This article starts from the view that a GND is positive/necessary and explores determinants of public support for such a programme in a context in which multiple factors tend towards the underestimation of climate risk/urgency of action. In particular, they propose that policy narratives developed by groups who oppose relevant policies will be persuasive.  The article finds that redistribution is the most persuasive narrative, which is seen as further evidence that material interest is a key determinant of policy preference, but that the GND was very popular already. 

 I am not qualified to comment on the statistical aspects of the paper, which is it majority. However, I have a few reflections that may strengthen the overall clarity/impact of its conclusions.

 1. The finding that material interest is a key determinant of policy preference counters increasing emphasis on "culture wars" in some corners. This point could be made more explicit and discussed head on.

 2. The overall aim of the paper comes across as a bit confused - the move to narrative development suggests its about how to persuade voters to support a GND, but the authors also find that support was high already (begging the question of why isn't government doing it if its so popular, but the paper doesn't address that question)

 3. Are there tensions/contradictions/other undesirable implications of the different narratives? For example, the energy security narrative may imply a nationalistic approach to a GND that is at odds with justice at an international scale

Is the work clearly and accurately presented and does it cite the current literature?

Yes

If applicable, is the statistical analysis and its interpretation appropriate?

I cannot comment. A qualified statistician is required.

Are all the source data underlying the results available to ensure full reproducibility?

Yes

Is the study design appropriate and is the work technically sound?

Yes

Are the conclusions drawn adequately supported by the results?

Yes

Are sufficient details of methods and analysis provided to allow replication by others?

Yes

Reviewer Expertise:

Political economy, sustinability transitions

I confirm that I have read this submission and believe that I have an appropriate level of expertise to confirm that it is of an acceptable scientific standard, however I have significant reservations, as outlined above.

# Response

**Published:** 2026-01-02

**Authors:** Matthew Johnson

**Affiliations:** Social Work, Education and Com, Northumbria University Department of Social Work Education and Community Wellbeing, Newcastle upon Tyne, England, UK

**Many thanks for this substantive review, which has been extremely helpful. In addition to the specific revisions below, we have also rewritten the conclusion.**

 1. The finding that material interest is a key determinant of policy preference counters increasing emphasis on "culture wars" in some corners. This point could be made more explicit and discussed head on.

**Response: Many thanks for this suggestion. We have added substantive discussion in the discussion section.**

 2. The overall aim of the paper comes across as a bit confused - the move to narrative development suggests its about how to persuade voters to support a GND, but the authors also find that support was high already (begging the question of why isn't government doing it if its so popular, but the paper doesn't address that question)

**Response: Many thanks for this suggestion. We have reframed the introduction, substantively enhancing discussion of the literature on firm opponents and their outsized role in stifling policy.**

 3. Are there tensions/contradictions/other undesirable implications of the different narratives? For example, the energy security narrative may imply a nationalistic approach to a GND that is at odds with justice at an international scale

**Response: Many thanks for this suggestion. We have added a note with regard to this at the end of the discussion.**

# Reviewer report: Reviewer response for version 1

**DOI:** 10.5256/f1000research.176615.r415860

**Published:** 2025-10-01

**Authors:** Andrew Roe-Crines

**Affiliations:** 1 University of Liverpool, Liverpool, UK

This article evaluates the attitude of the Labour Government towards Climate Change and promised investment in order to tackle the consequences of the changing environmental behaviour. The article rightly notes the urgency of needed action, yet the Labour Government has essentially failed to rise to the challenge of providing leadership and resources. The rationale for the failure is to highlight the priority of the economy and seeking to restore confidence in the free-market economy following the consequences of COVID and the shutting down of many businesses for a sustained period.

 The article benefits from an interesting survey of the public, thereby offering an original analysis of public opinion, alongside a distinctive methodological approach. Indeed, the mixed-methods enables the article to look at both statistical data qualitative perspectives, thus giving the article an essential sense of depth to the range of research. I would contend that the article is thoroughly researched, which enables the author(s) to produce a series of evidence-based conclusions. The statistical models used by the author(s) to demonstrate the levels of appeal are valuable demonstrations of opinion across the main political parties. Indeed, the levels of support for particular political parties impacts on the formulation of public policy on a range of issues, including Climate Change. It is important, however to bear in mind that there remains opponents to climate change action from the left and right, alongside support for action coming from Conservatives as well as Liberal Social Democrats. Indeed, speaking to the United Nations in 1989, Mrs Thatcher argued that “what we are now doing to the world, by degrading the land surfaces, by polluting the waters and by adding greenhouse gases to the air at an unprecedented rate - all this is new in the experience of the earth. It is mankind and his activities that are changing the environment of our planet in damaging and dangerous ways” (Thatcher, 1989). To address this problem, she called of international cooperation, saying “it is no good squabbling over who is responsible or who should pay. We shall only succeed in dealing with the problems through a vast international, co-operative effort” ( *ibid.).* This demonstrates the longevity of the issue, and the calls for unified action as long ago as Mrs Thatcher’s administration – yet, despite this and current voter attitudes, the action Mrs Thatcher called for remains elusive, much to the detriment of environment.

 The conclusions of the article demonstrate how substantive reforms of climate change action via the Green New Deal would improve economic performance of the UK overall. Needless to say, this would necessitate a collegiate working relationship between employers, employees, wider population, and the government regulator/investor. Such an alignment is, however some distance from where we are, given the priorities of each group can be substantially different. For example, employers prioritise business sustainability and maximising profiles; employees exchange their labour for renumeration; the wider population is concerned by the growing danger posed by unaddressed Climate Change; and the government priorities include economic management, social policy, foreign policy, alongside wider issues such as healthcare and law and order. This demonstrates the incompatibility of priorities, despite the need to come together in order to address a global crisis which necessitates unified public policy action.

 On the whole, this was a very well researched and evidence-based article which presents valuable findings on public attitudes and a Green New Dea. Indeed, the article presents an important set of findings concerning an issue that transcends border, political parties, ideologies, and economic systems. Needless to say, opposition from this approach will come from a number of national and international groups, especially those who already receive the greatest financial benefits from the status quo. Yet the power of the government can be used to incentivise business through innovations such as tax-breaks for those embracing a Green New Deal, as well as imposing penalties for a lack of action.

 The innovative distinctiveness of the article comes from the presentation of a Green New Deal draft and the corresponding views of the wider public. By doing so, the article contributes a significant series of valuable statistical findings, thereby demonstrating the levels of potential appeal should the government embrace Green New Deal.  In my view, this is an insightful, interesting, and valuable article which demonstrates the political and intellectual significance of Climate Change measures, alongside the depths of support across the main political party support-bases.

 Cited Quote:

 Thatcher, M. (1989) ‘Margaret Thatcher: How the PM legitimised free concerns’, *BBC News*. Available from: https://www.bbc.co.uk/news/science-environment-22069768.

Is the work clearly and accurately presented and does it cite the current literature?

Yes

If applicable, is the statistical analysis and its interpretation appropriate?

Yes

Are all the source data underlying the results available to ensure full reproducibility?

Yes

Is the study design appropriate and is the work technically sound?

Yes

Are the conclusions drawn adequately supported by the results?

Yes

Are sufficient details of methods and analysis provided to allow replication by others?

Yes

Reviewer Expertise:

Political Communication; British Politics; Voting Behaviour; Ideologies; Institutions.

I confirm that I have read this submission and believe that I have an appropriate level of expertise to confirm that it is of an acceptable scientific standard.

# Response

**Published:** 2026-01-02

**Authors:** Matthew Johnson

**Affiliations:** Social Work, Education and Com, Northumbria University Department of Social Work Education and Community Wellbeing, Newcastle upon Tyne, England, UK

We are extremely grateful for this careful and expansive reading and engagement with our article. We were particularly taken by reference to Mrs Thatcher’s concern for climate change mitigation in 1989 and have referenced this as evidence of the possibility of non-progressive parties’ supporting a Green New Deal if sufficient electoral feasibility is demonstrated. Many thanks for your engagement.
